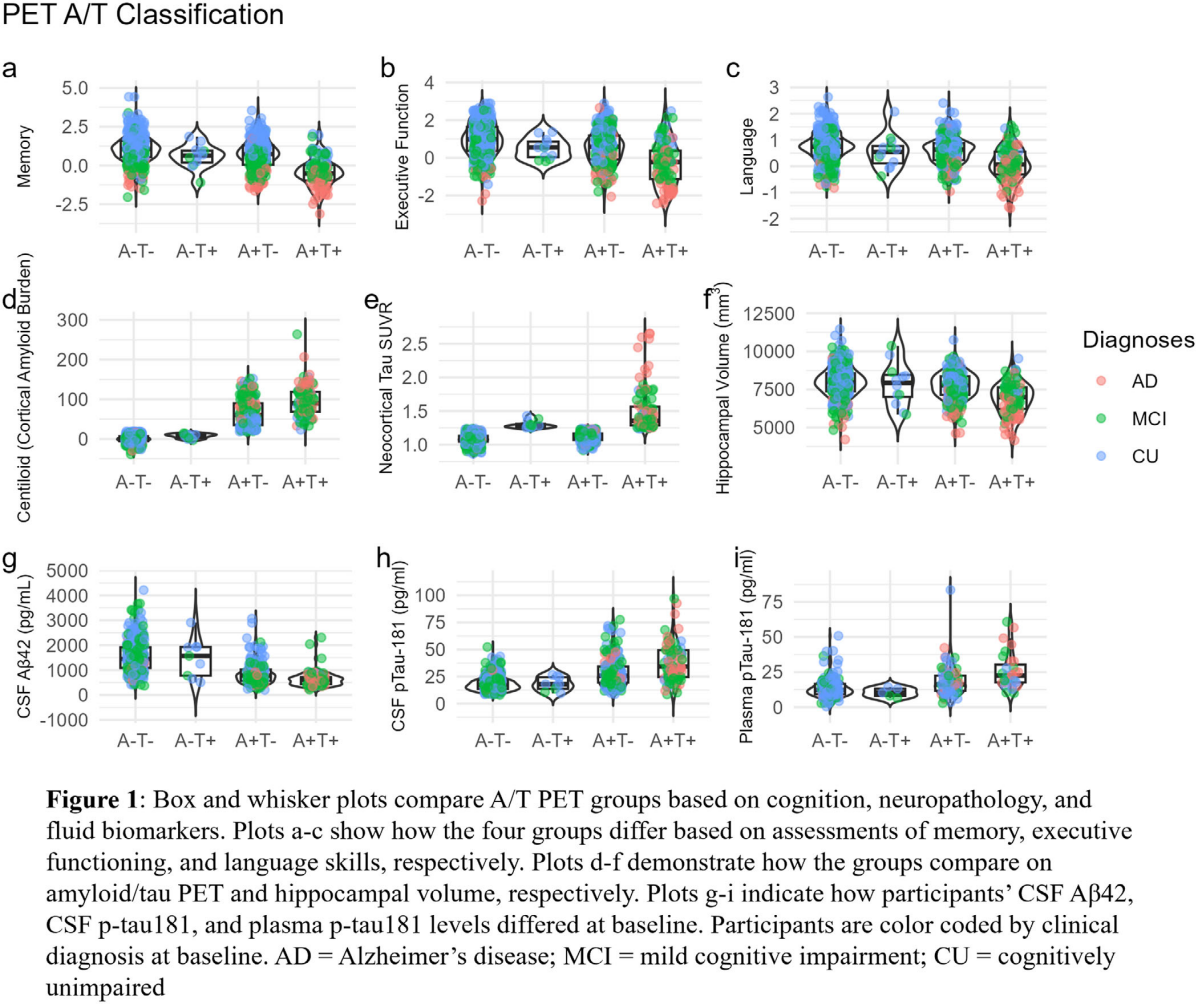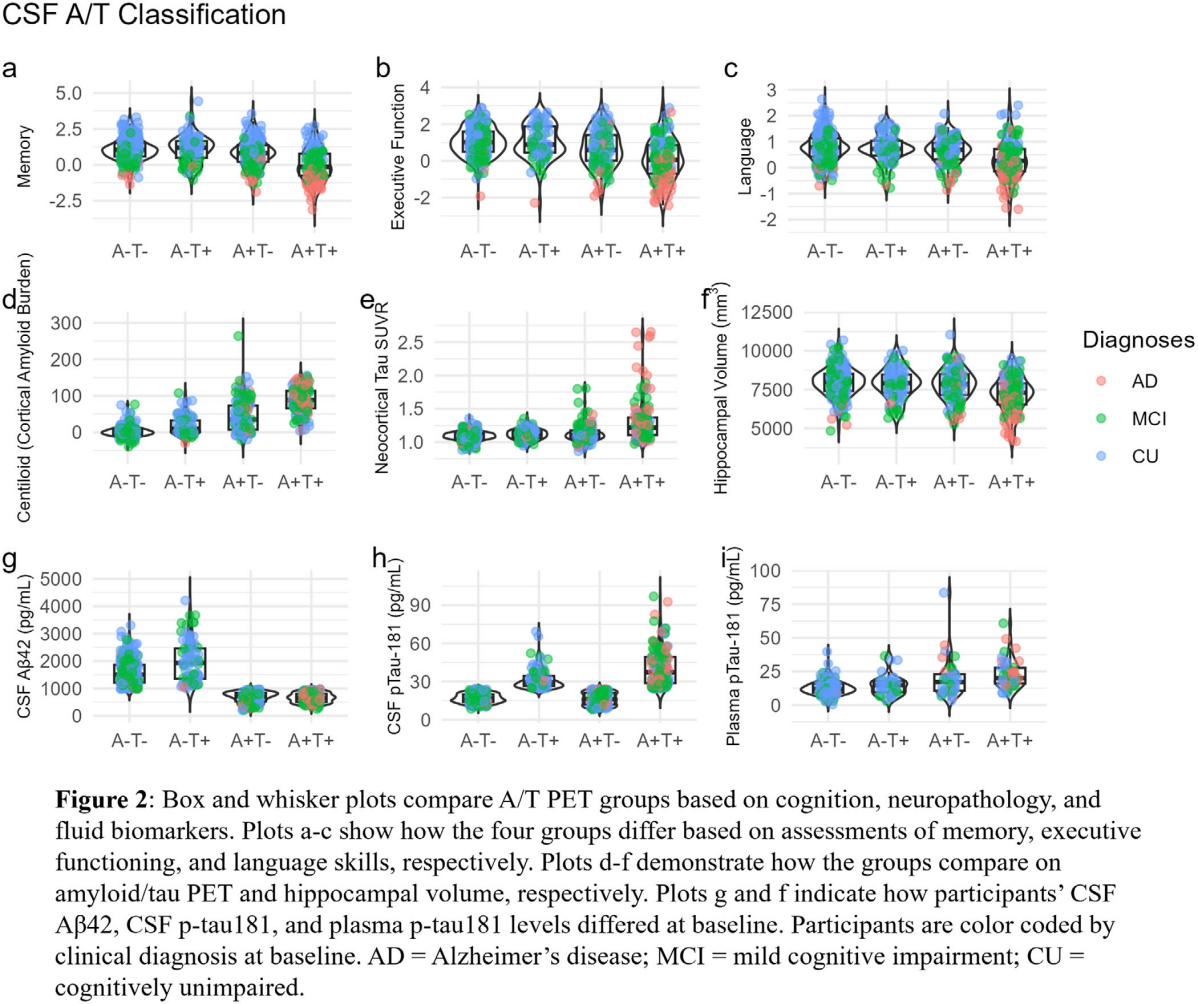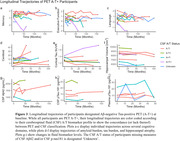# Characterizing A‐T+ PET participants across the Alzheimer’s disease spectrum in the ADNI cohort

**DOI:** 10.1002/alz.095514

**Published:** 2025-01-09

**Authors:** Daniel C Bowie, Yara Yakoub, Sylvia Villeneuve, Alzheimer’s Disease Neuroimaging Initiative

**Affiliations:** ^1^ McGill University, Montreal, QC Canada; ^2^ Douglas Mental Health University Institute, Centre for Studies on the Prevention of Alzheimer’s Disease (StoP‐AD), Montréal, QC Canada; ^3^ Integrated Program in Neurosciences, McGill University, Montreal, QC Canada

## Abstract

**Background:**

For individuals on the Alzheimer’s disease trajectory, amyloid positivity generally precedes tau positivity, as defined by PET biomarkers. However, multiple studies report amyloid‐negative tau‐positive PET (A‐T+) participants, whose clinical implications remain unclear. Here, utilizing the Alzheimer’s Disease Neuroimaging Initiative (ADNI) cohort, we examined how A‐T+ participants differ from other A/T profiles based on cognition, fluid biomarkers, and neuropathology—we also investigated the longitudinal trajectories of A‐T+ participants.

**Method:**

We studied 806 participants from the ADNI cohort who underwent an initial amyloid (^18^F‐Florbetaben, FBB, or ^18^F‐Florbetapir, FBP) and tau (^18^F‐Flortaucipir, FTP) PET‐scan. The amyloid‐positivity thresholds for FBB and FBP were SUVR = 1.08 and SUVR = 1.11, respectively. Tau‐positivity was set as 2SD above the mean of Aβ‐PET negative CU participants in a neocortical ROI (SUVR = 1.24). The A‐T+ participants were then compared to other A/T biomarker groups in terms of clinical diagnoses, CSF/plasma Aβ and ptau, and hippocampal volume. Using a similar approach, we assessed the concordance of A‐T+ participants using CSF Aβ42 (981 pg/mL) and phosphorylated tau 181 (24.3 pg/mL) instead. Finally, we assessed the cognitive and biomarker trajectories of PET A‐T+ participants.

**Result:**

Twelve participants were classified as PET A‐T+. Of eleven PET A‐T+ participants with CSF biomarker data, only two (18%) were also CSF A‐T+ (A‐T‐ = 4, A+T‐ = 2, A+T+ = 3). Across all outcomes, A‐T+ (PET or CSF) participants fared similarly to A‐T‐ participants (**Figures 1 and 2**). Overall, PET A‐T+ exhibited benign longitudinal trajectories (**Figure 3**). However, one participant, classified as PET A‐T+ but CSF A+T+, displayed concomitant precipitous declines across all cognitive domains, which coincided with an acceleration in neocortical tau burden.

**Conclusion:**

While displaying pathological levels of cerebral tau, PET A‐T+ participants are highly similarly to PET A‐T‐ participants. Additionally, there is minimal overlap between PET A‐T+ and CSF A‐T+ participants, emphasizing the fact that PET and CSF biomarkers are not interchangeable. While A‐T+ may reflect participants with primary age‐related tauopathy (PART), in which amyloidosis is not the primary driver of tau accumulation, some A‐T+ participants might also have amyloidosis levels that are not yet detectable with PET.